# Are CT Scans a Satisfactory Substitute for the Follow-Up of RSA Migration Studies of Uncemented Cups? A Comparison of RSA Double Examinations and CT Datasets of 46 Total Hip Arthroplasties

**DOI:** 10.1155/2017/3681458

**Published:** 2017-01-24

**Authors:** Volker Otten, Gerald Q. Maguire Jr., Marilyn E. Noz, Michael P. Zeleznik, Kjell G. Nilsson, Henrik Olivecrona

**Affiliations:** ^1^Department of Surgical and Perioperative Sciences, Umeå University, Umeå, Sweden; ^2^School of Information and Communication Technology, KTH Royal Institute of Technology, Kista, Sweden; ^3^Department of Radiology, New York University School of Medicine, New York, NY, USA; ^4^School of Computing, College of Engineering, University of Utah, Salt Lake City, UT, USA; ^5^Department of Molecular Medicine and Surgery, Karolinska Institutet, Stockholm, Sweden

## Abstract

As part of the 14-year follow-up of a prospectively randomized radiostereometry (RSA) study on uncemented cup fixation, two pairs of stereo radiographs and a CT scan of 46 hips were compared. Tantalum beads, inserted during the primary operation, were detected in the CT volume and the stereo radiographs and used to produce datasets of 3D coordinates. The limit of agreement between the combined CT and RSA datasets was calculated in the same way as the precision of the double RSA examination. The precision of RSA corresponding to the 99% confidence interval was 1.36°, 1.36°, and 0.60° for *X*-, *Y*-, and *Z*-rotation and 0.40, 0.17, and 0.37 mm for *X*-, *Y*-, and *Z*-translation. The limit of agreement between CT and RSA was 1.51°, 2.17°, and 1.05° for rotation and 0.59, 0.56, and 0.74 mm for translation. The differences between CT and RSA are close to the described normal 99% confidence interval for precision in RSA: 0.3° to 2° for rotation and 0.15 to 0.6 mm for translation. We conclude that measurements using CT and RSA are comparable and that CT can be used for migration studies for longitudinal evaluations of patients with RSA markers.

## 1. Introduction

Reliable measurement of implant migration enables early detection of an inferiorly performing prosthesis as well as identifying patients in whom aseptic loosening may occur sooner than expected [1, 2]. Radiostereometry (RSA), introduced in 1974 [3], is considered the gold standard for evaluation of implant migration in vivo [4, 5]. In many patients (roughly 10,000 in Sweden alone [6]) tantalum beads were implanted into the bone and attached to or inserted into the prosthesis for assessment of joint replacements with RSA. However, RSA requires calibration cages, radiology facilities with two X-ray machines for simultaneous acquisition of two X-ray images, specialized software, and trained personnel [7]. The number of hospitals capable of this analysis is limited; thus alternative techniques, for following these patients over time, such as three-dimensional (3D) computed tomography (CT), should be considered. Today, CT-scanners are found in almost every hospital and these scanners make it possible to visualize the small tantalum beads (RSA markers) in CT volumes with a clinically acceptable accuracy of 1 mm or less [8–10]. Additionally, the effective radiation dose of CT scans is constantly being lowered and the quality of the scans with regard to metal artifacts is improving [11]. If 3D CT is comparable in precision to RSA, then CT could replace RSA, thus greatly expanding the cohort of patients who could be followed.

Over the last decade, our group has refined and validated a 3D volumetric data processing tool [12–15], which exploits cohomologous points (landmarks) to enable volume fusion. This tool has been used with 3D CT volumes for studying the position, wear, and migration of orthopedic implants over time. This report explores the possibility of utilizing CT scans of patients with tantalum beads in the acetabulum and orthopedic hip implants, by directly comparing CT derived data to RSA data acquired on the same day. Specifically, we evaluate how similar a dataset of 3D bead positions derived from routine CT scans of patients is to a RSA dataset based upon these same RSA markers. If sufficiently similar, then CT could be used to follow patients from RSA migration studies, for example, to evaluate new prostheses [10].

## 2. Materials and Methods

### 2.1. Patients

The data for this study resulted from a 14-year follow-up of total hip arthroplasty [21] whose purpose was to investigate the influence of different cup augmentation on cup stability and liner wear. Sixty-three patients (68 hips: 31 men, median age (range) 56 (36–66) years, median weight (range) 75 (51–102) kg) with primary and secondary hip osteoarthritis (OA) underwent total hip arthroplasty between 1995 and 1997 at the Department of Orthopedics, Umeå University Hospital. Fifty-two hips had primary and 16 had secondary OA with 37 hips in Charnley class A (unilateral OA) and 31 hips in class B (bilateral OA) [22]. A hemispherical porous-coated titanium alloy cup with ethylene oxide sterilized polyethylene liner (Reflection, Smith & Nephew, Memphis, TN) was used in all patients. During surgery, five to nine 0.8 mm tantalum beads were inserted into both the periphery of the polyethylene liner and the acetabular bone as RSA markers.

At the 5-year follow-up [23] a high amount of wear of the polyethylene liner, which increases the risk of osteolysis development over time [24], was evident. Therefore, a CT scan was added for the 14-year follow-up to investigate the amount of osteolysis around the cup. This enabled comparing the 3D RSA marker positions (landmarks) derived from CT with those provided by RSA. The institutional review board approved the study (the Central Ethical Review Board of Umeå University Dnr 2010-100-31) and all patients gave their written consent.

At the 14-year follow-up 48 hips in 45 patients remained and were included in this study. Reasons for not attending the follow-up were death (8 patients, 9 hips), revision (5 patients, 6 hips), or refusal to attend (5 patients, 5 hips). The median (range) follow-up time was 14 (13–15) years. Clinical outcome and results of the migration measurements were published in a separate paper [21]. Each patient had a CT scan and 2–4 stereo radiograph pair examinations (resulting in 4–8 individual X-rays) on the same day.

### 2.2. CT Scans

CT scans (LightSpeed VCT General Electric Medical Systems, Milwaukee, WI) were acquired from spina iliaca anterior superior to a point at least two centimeters distal to the tip of the femoral component with scan parameters: data collection diameter: 500 mm, reconstruction diameter: 400 mm, source to detector distance: 949.08 mm, source to patient distance: 541 mm, peak kilovoltage (kVp): 120 kV, and exposure time: 800 ms. A bone reconstruction algorithm was used resulting in 225 to 366 slices at 0.625 mm increments with a matrix size of 512 × 512 and a pixel resolution of 0.78 × 0.78 mm in the axial (*xy*)-plane. The median (range) effective radiation dose was 6.9  (3.2–21.1)  millisievert  (mSv) [25]. To facilitate analysis of both CT and RSA derived data, the CT volume for each patient was duplicated and given a new name.

### 2.3. Radiostereometry (RSA)

A ceiling stand (Aristos VX, Siemens, Munich, Germany) was combined with a mobile digital X-ray unit (Mobilett, Siemens, Munich, Germany), both having a resolution of 10 pixels per mm to obtain two simultaneous exposures of the hip joint. Patients were placed supine on the examination table with the planes of the two X-ray tubes angled by approximately 40° to each other combined with a uniplanar calibration cage No. 43 (RSA Biomedical, Umeå, Sweden). Two image pairs (a double examination: 4 X-ray images) were obtained with slightly different patient positions. In some cases an additional one or two extra image pairs were needed until two image pairs with optimal visualization of the tantalum markers on both foci were achieved. The median (range) effective radiation dose per complete examination, calculated using PCXMC version 1.0 [26], was 1.9 (1.9–3.8) mSv for a fully digitized acquisition at 120 kV and 150 mAs.

### 2.4. RSA Procedure

The radiographs were analyzed using the UmRSA software 6.0 (RSA Biomedical AB, Umeå, Sweden) by a skilled operator with many years' experience. All tantalum beads (markers) that could be identified on both focuses 1 and 2 images were given a unique marker number. The 3D coordinates (in the standardized RSA cage coordinate system) for the markers were calculated, where the medial-lateral position is described on the *x*-axis, distal-proximal on the *y*-axis, and posterior-anterior on the *z*-axis [27]. The following data were saved for later analysis: focuses 1 and 2 stereo radiograph images (each showing the markers in the periacetabular skeleton and in the polyethylene liner with corresponding marker numbers) and a dataset consisting of marker number and 3D coordinates for this marker.

The precision of the current RSA setup was calculated by double examinations of all 48 hips. Markers in both the acetabulum and the rim of the liner were accepted when the mean error of rigid body fitting was ≤0.25 mm with condition number (reflecting how well spread the markers are in 3D) < 90, or ≤0.35 mm with condition number <80, or ≤0.2 mm with condition number ≤130. Markers that did not meet these criteria were automatically excluded assuming instability of the marker or incorrect marking, resulting in 46 RSA double examinations (46 hips, 43 patients) for analysis.

### 2.5. Data Processing

The RSA coordinate system is determined by the reference cage, where normally for the first set of postoperative radiographs at least one set is acquired with the patient carefully oriented relative to the external reference cage [18]. In contrast, the orientation of the patient during the CT scan is unimportant because the volume can be freely rotated in 3D [15].

By designating landmarks in the CT volume (i.e., defining a landmark's 3D coordinates) and transforming the coordinates of the RSA markers from the RSA coordinate system into the CT coordinate system, we were able to treat the RSA dataset and the CT dataset as a double examination; hence we compared the limit of agreement (precision) of this calculation with that from a RSA double examination. For calculating the limit of agreement always the first of the two RSA image pairs was used.

The analysis of the CT data to produce the 3D CT datasets and the comparison of these datasets with the RSA datasets were both performed using a previously described and validated 3D volume fusion (spatial registration) tool [12, 28, 29]. This semiautomated tool provides landmark-based fusion of two volumes, registering a “target” volume to a “reference” volume by exploiting cohomologous landmarks in each volume, via a variety of 3D transform modules, ranging from simple rigid body to 3D warping and including user-defined transformations. A technical description can be found in earlier publications [12, 14, 30]. Moreover, use of the CT volumes gives us the opportunity to work directly in three dimensions, while tracking each step graphically.

### 2.6. CT-Based Procedure

**Figure 1:**
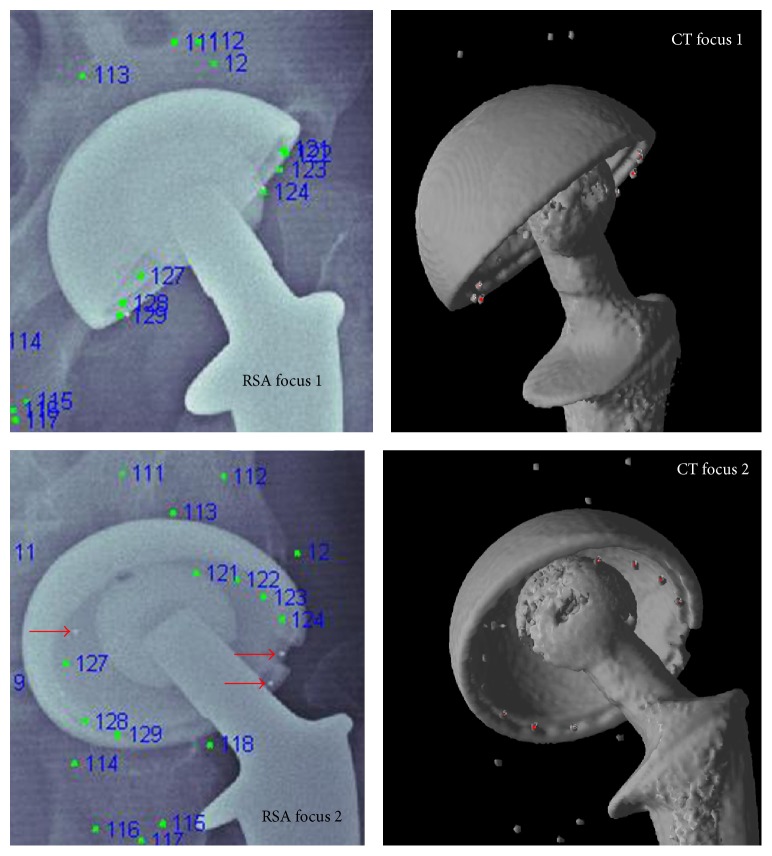
The isosurface level for the CT volumes is set so that only metal is visible. The image of the 3D CT volume is rotated until it matches focuses 1 and 2 of the RSA radiographs. The tantalum beads in the periacetabular bone are numbered in the CT volume in the same sequence as in the RSA radiographs. Via rigid body transformation these bone marker coordinates from the RSA volume and CT volume are matched. Thereafter the tantalum beads of the liner in the CT volume are marked in the same way. Markers visible in only one of the RSA foci (red arrows) are excluded even in the CT volume.

First the tantalum beads were visualized in the CT volume by setting the isosurface level such that voxels attenuating less than metal were not visible. Then, in order to assign landmark numbers consistent with the RSA marker numbers, in a separate display the RSA focuses 1 and 2 stereo radiograph images showing the tantalum beads with their marker numbers were displayed. The CT volumes were manually rotated in 3D so they were visually comparable with the stereo radiographs. Using the computer's pointing device, the tantalum beads in the bone were designated and assigned the same marker numbers as in RSA (Figure 1). When a bead was designated, the fusion tool automatically found a best fit center and recorded this as a 3D landmark (see in particular Figure  2 in [10] which shows that even in the presence of partial volume effects the landmark is visually embedded centrally in the volume of the bead). This created a set of “CT-based bone landmarks” in the CT data coordinate system.

To utilize the RSA dataset, the fusion tool mapped the RSA cage coordinate system into the CT data coordinate system. This transformation process was previously described and validated to have a median (range) translation error of 0.22 (0.07–0.52) mm, a rotation error of 0.003 (0.001–0.006)°, and a goodness of fit of 0.0010 (0.0001–0.022) per bead [9]. We then imported and transformed the RSA bone marker dataset into a set of “RSA-based bone landmarks” in the CT coordinate system. This produced two CT data volumes: one containing bone landmarks generated from 3D CT analysis and the other containing bone landmarks generated from RSA (transformed into the CT coordinate system).

The CT volume containing the “RSA-based bone landmarks” was used as the “reference” volume, while the CT volume containing the “CT-based bone landmarks” was used as the “target” volume. The fusion tool then registered the target volume with the reference volume using corresponding landmarks. The result was a rigid body transformation that registers the two CT volumes based upon the entire set of “CT-based bone landmarks” and “RSA-based bone landmarks” using singular value decomposition (SVD) [31]. The distances between each of the original landmarks of the reference volume and the new (transformed) landmarks of the target volume were automatically generated.

Because the “RSA-based bone landmarks” used were derived from the original RSA dataset* before* the automated process where the RSA software deletes misplaced points or performs any “trial and error” interchanging of marker numbers, the set contains both correct points and incorrect points (i.e., those where the coordinates of a marker were derived from two different markers or from a marker and background noise, such as the femoral head or the acetabular shell). Corresponding landmark pairs that were not well matched (i.e., those whose distance is different from the corresponding landmark by ≥2.0 mm) were manually interchanged or excluded by the operator until a reasonable (i.e., the maximum 3D distance error of any landmark pair was ≤2.0 mm) registration between the two volumes based on “bone landmark” sets was established.

The landmark selection procedure was then repeated for the “prosthesis” landmarks instead of the “bone” landmarks. This resulted in a corresponding set of “RSA-based prosthetic landmarks” in the “reference” volume and “CT-based prosthetic landmarks" in the “target” volume. A rigid body transformation that registered the entire set of “CT-based prosthetic landmarks” to the entire set of “RSA prosthetic landmarks” was then performed, followed by the verification of the landmark selection procedure.

In summary, 6 steps are necessary to calculate the error of the cup position in relation to the acetabulum bone between CT and RSA: (1) assigning bone and prosthetic markers in CT consistent with the marker numbers in RSA, (2) importing landmark coordinates from RSA into a copy of the CT volume, (3) registering the target volume (CT) to the reference volume (RSA) based on the bone landmark sets using a rigid body transformation, (4) transforming the target prosthetic landmark set into the registered coordinate system, (5) rotating and translating both volumes and associated prosthetic landmarks to a standard orientation, and finally (6) computing the rigid body transformation that would move the resulting target prosthetic landmark sets into spatial alignment with the reference set [30]. Steps (1) and (2) were done manually, while steps (3) to (6) were performed automatically.

The rotation point for the last rigid body transformation is by default the centroid (the geometric weight-point) of the prosthetic reference landmark set. Using the SVD registration method, the program computes this transformation as a 3 × 3 rotation matrix and a 1 × 3 translation matrix in the CT coordinate system. The main outputs from this process are (1) visual 2D and 3D images of the two volumes after registration of the bone landmark set and (2) numerical data indicating the movement of the prosthesis in six degrees of freedom (rotations about and translations along the *x*-, *y*-, and *z*-axes, where the translations are given for each individual prosthetic landmark with respect to the centroid of the prosthetic landmarks). The error of the measurement is the last registration movement between the rigid body from RSA dataset and CT dataset. The rotation errors are generated by decomposing the rotation matrix into Euler angles, which, although not unique, are computed as a clockwise rotation first around the *x*-, then the *y*-, and finally the *z*-axes as defined by the standardized RSA in the CT coordinate system. Numerical information, based on the distance between the original landmarks of the stationary body and the new (transformed) locations of the moved body for each landmark, for both the bone and the prosthetic landmarks is automatically generated. These differences are computed for each orthogonal direction, for the axial (*xy*)-plane and for the volume. If the registrations were perfect, these differences would be zero. For a more detailed description of this method, see the previous study [30].

### 2.7. Evaluation of Errors

The error in the bone and prosthetic landmark sets was expressed as the differences between the RSA and the CT-based coordinates of the markers. In the 3D view of the CT volume, it is easy to determine if the marking of the tantalum beads from RSA is correct. Hence, in contrast to the analysis of the RSA double examinations, we did not set a maximum value for the mean error but rather simply excluded those markers that, on visual inspection, could be seen to be incorrectly marked or not marked at all in the RSA setup.

The relative movement of the prosthetic landmark group after the final transformation was expressed in 6 degrees of freedom (Euler angles and translation distances). All of the prostheses were considered to be stable in the bone [21]; therefore, the movement of the prosthesis landmarks between RSA and CT should ideally be zero.

The limit of agreement was calculated as mean and median of the differences for both rotation and translation plus and minus two standard deviations [32]. This was acceptable as the data was determined to nearly follow a normal (Gaussian) distribution using histograms, box, density, and quantile-quantile plots [33]. The mean differences ± 1.96 *∗* standard deviation is the 95% confidence interval [34]. Additionally, we calculate the maximum total point motion (MTPM), that is, the length of the 3D vector of the marker that moved the most. Since calculation of the limit of agreement has been performed in several different ways in other studies, we also calculated the results as mean ± 2.01 (*t* statistic for 46 degrees of freedom) *∗* standard deviation giving the 95% quantile for 46 double examinations [35]. A third way of presenting this data has been frequently used in earlier RSA studies [5, 23, 36, 37] with the 95% confidence limit expressed as the mean of the absolute differences plus 1.96 *∗* standard deviation of the differences (and plus 2.575 *∗* standard deviation for the 99% confidence limit). The density plots shown, which express the frequency distribution between the first and third quantiles of the date, were calculated using Gaussian filter. The statistical program R version 3.1 was used [33].

## 3. Results

All markers designated on the radiographs could be visualized in the CT volumes. Further, a consistent finding was that additional markers were clearly identified in the CT volumes, which had not been designated on the stereo radiographs. Only markers that could be clearly identified on both focuses 1 and 2 of the stereo radiographs were used in the analysis. Of the excluded markers, one prosthetic marker that had been correctly designated on radiographs was excluded due to bad marking on CT. This marker was located less than one mm from the cup shell, and information from the cup interacted in the automated process, resulting in off-centering of the landmark. The remaining excluded markers were excluded due to being incorrectly marked on the radiographs.

**Figure 2:**
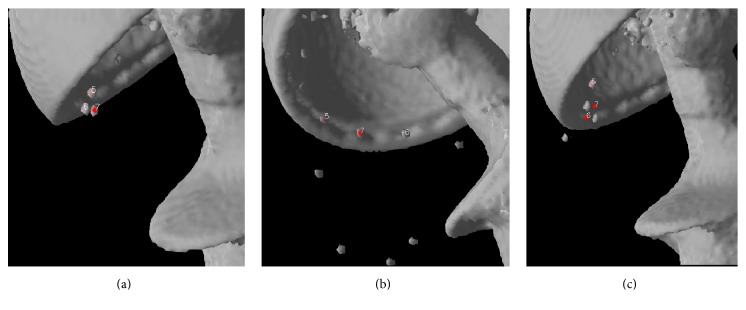
The process of eliminating inappropriate landmarks is illustrated. Two landmarks derived from the RSA data file are visualized in the CT volume. The CT volume can be freely rotated. In the rotation corresponding to focuses 1 (a) and 2 (b), the landmarks seem to correspond to the marker beads. Rotation of the 3D images of the CT volume reveals that the points have been interchanged on the RSA radiographs (c).

All 48 hips could be registered with respect to the tantalum beads in the acetabulum with a median (range) of eight (4–9) landmarks. After registration, a majority of the landmarks showed an overlapping pattern, with the center of all RSA derived landmarks placed inside the volume of the tantalum bead representation in the 3D CT volumes (Figure 1). In two patients, there were only two valid landmarks from the stereographs in the cup, leaving 46 hips for registering the cups and computing relative movement. The remaining cup landmark sets had a median (range) of five (3–9) landmarks. Figures 2(a)–2(c) show the process for identifying markers that had been inappropriately designated as being the same.

**Figure 3:**
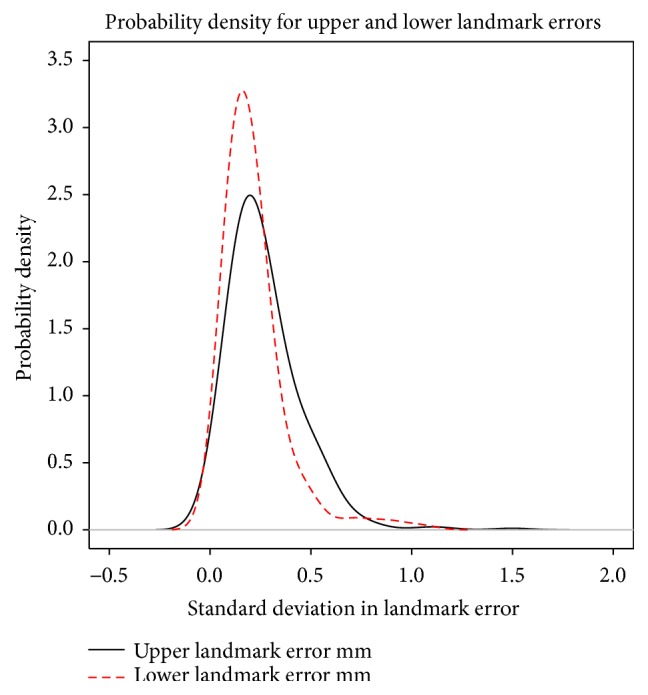
Probability density plot of errors of rigid body fitting describing the relative likelihood for the distance between individual RSA and CT derived landmarks to take on a given value after rigid body fitting. The probability of the distance falling within a particular range of values is given by the area under the density function.

The errors of individual markers after rigid body registration for both bone and prosthetic landmarks are given in Table 1 both for the RSA double examinations and between CT and RSA. For the RSA double examinations all markers with a mean error above 0.35 mm were excluded in the UmRSA software. Density plots for the landmark errors are given in Figure 3. The data were close to normal. Note that there are some outliers. A subanalysis of landmarks with large errors showed that several of these were “double balls,” that is, two tantalum beads simultaneously in close proximity. The RSA images separated these quite effectively, but our automated routine tended to push the two CT designated landmarks together due to interference. This combination gives a pair of landmarks with somewhat higher error after rigid body registration, but the combined effect on the evaluation of relative movement is theoretically negligible, since these errors tend to eliminate each other.

**Figure 4:**
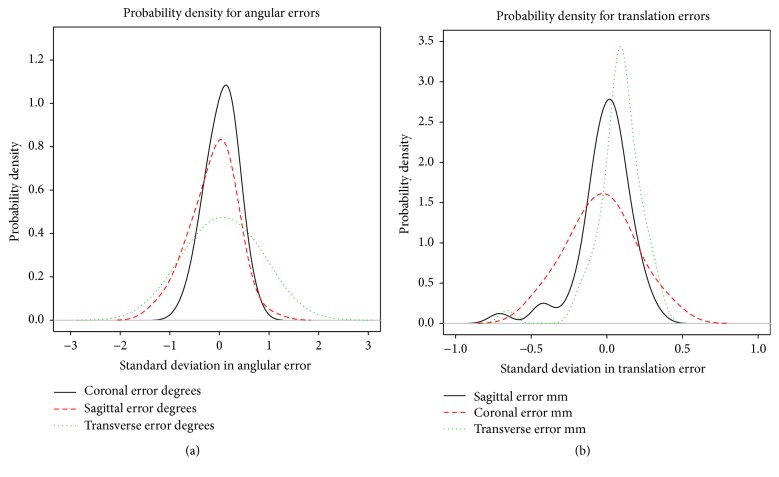
Probability density plot of errors of measurement of relative movement.

Analysis of relative movement, presenting the precision of our setup, is given in Tables 2(a)–2(c).  Figure 4 shows the density plots for errors in the rotations and translations.

## 4. Discussion

From the user's standpoint, marking the volumes was easier and faster when using CT than RSA. In addition, in contrast to radiographs, where markers are often shadowed by the prosthesis, we could consistently find all markers in the CT volumes. For this study, we performed the analysis of component movement directly in the program developed for the CT volumes. Theoretically, it would also be possible to reverse the registration process between CT and RSA derived dataset, then importing the CT coordinate dataset into the existing RSA analysis software and performing the subsequent analysis using that software.

A key feature of the UmRSA software is its calculation of mean error of rigid body fitting (ME). This reflects the relative motion of individual markers in each segment and the error of digitization between two different examinations and thus is a measure of the stability of the rigid body. In clinical RSA trials, ME values of 0.10–0.25 mm are typical [5] when using commercially available software (e.g., UmRSA 6.0, RSA Biomedical, Umeå, Sweden). Increasing ME decreased the precision of RSA in a laboratory study [38]. At the start of a normal RSA analysis, the user defines an upper limit for acceptable individual landmark error; then points outside of this limit are automatically excluded during subsequent calculations. In this study, we transformed the CT volumes into the RSA derived landmarks and then manually excluded points that were obviously misplaced during the marking of the radiographs (Figure 2). Therefore, the mean errors found in this study differ from the standard ME values, because all points that are accepted as being cohomologous are included. In addition, the automated routine in the program that finds the centroid of the tantalum beads in the CT dataset is not optimized for beads in close vicinity to another metal object, as was the case for double markers and markers less than a millimeter from the cup shell. For bone markers or markers placed in the liner this is rarely a problem. When markers are attached directly to the metal of the prosthesis it might be an issue. This can be addressed by changing parameters in the program or by manual designation of the marker's centroid [9]. However, in this study the aim was to evaluate how similar a RSA dataset and a dataset derived from routine CT scans of patients would be, when essentially everything but designating the marker sequence was calculated by the program. Slightly higher MEs were expected and also that these errors would be generated from both input modalities. The MEs of rigid body registration between the RSA and CT volumes in this study are of the same order of magnitude as ME for clinical RSA trials, indicating that, from a standpoint of quality of input data, it would be feasible to continue monitoring patients with tantalum beads. It might be possible to start new migration studies using CT if one lacks a facility for RSA radiographs [16, 29]. As stated in the introduction, the effective radiation dose of CT scans is constantly being lowered and the quality of the scans with regard to metal artifacts is improving [11]. The precision of RSA in a clinical setting, based on double examinations, has been shown to vary between 0.15 and 0.60 mm for translations and between 0.3 and 2° for rotations at a 99% confidence limit [5]. The agreement between the current CT and RSA datasets presented in this study as determined by multiple previously published methods is shown in Tables 2(a)–2(c).

From the standpoint of precision, treating the comparison between RSA and CT as a double examination, we find that this is comparable with RSA double examinations (Table 2(a)). Many factors influence the ability to perform RSA analyses in a negative way. However, with CT several of these factors can be avoided, such as the loss of markers because of shadowing by the implant, the varying position of the patient in relation to the cage, and the mismatching of markers in the different focal planes.

In the CT volume it was possible to identify more markers than were found in the pairs of RSA examination. Unfortunately, for this study we could not exploit this advantage because all markers not found via RSA were excluded. However, using all the available markers might give a more stable rigid body in the segments (bone or prosthesis) when following patients over time with CT.

To achieve the results reported here, using a standard bone reconstruction algorithm for the CT scan was sufficient. This opens the possibility of measuring migration in a precise way when CT examinations are performed because of other reasons on patients with implanted tantalum beads, especially if it is impossible to perform RSA because the patient has moved to a place where RSA acquisition is unavailable. This CT derived data could also potentially be used to augment the data available for long term studies of patients by providing datasets at different times between the scheduled *n*-year follow-up periods, thus providing data for patients who perhaps do not complete the full study.

From a standpoint of radiation exposure, the CT examinations used in this study did expose the patient to higher radiation levels than the RSA examinations. However, these CT volumes were primarily obtained to analyze osteolysis around the cup and were not optimized in any way for simply locating the tantalum beads. Several studies on the extremities and of the glenohumeral joint show that large acceptable effective radiation dose reductions can be obtained when imaging tantalum beads using CT without losing image quality [17, 19, 20]. A decade ago, Gurung et al. used a 16-row CT to study effective dose reduction in pelvic scans and showed that they could acquire scans at an effective radiation dose of 2.2 mSv which allowed valuation of the acetabulum and the iliosacral joints according to specific detailed criteria [39]. There is, to our knowledge, no study on dose reduction in patients when imaging tantalum beads in the hip. Theoretically, reasonable imaging of the tantalum beads in patients should be attainable at significant dose reductions, as has been shown in the shoulder and extremities. In a phantom study, where the precision and accuracy of the CT method is evaluated using double RSA examinations and double CT examinations, we have performed scans that, if applied to a patient, would give an effective dose of 0.4 mSv. In another study, this dose is further reduced in a porcine model [11]. Results from these two studies indicate that, under laboratory conditions, the effective radiation dose can be significantly reduced without significant loss of precision and CT's precision is comparable to RSA.

In previous publications, we showed that RSA data could be retrospectively registered to and visualized in CT volumes [9, 40] and can be used to follow patients with implanted tantalum beads [10]. In this study we demonstrate that when RSA data of clinical patients is registered to CT data, the agreement is sufficient that longitudinal or double CT exams could be used for evaluation of prosthetic movement.

## 5. Conclusions

CT scans can be used for migration measurements in longitudinal evaluations of patients with RSA markers without losing clinically significant precision. The use of CT scans enables following RSA studies started earlier using a normal CT scan, thus avoiding the need for special RSA radiology facilities.

## Figures and Tables

**Table 1:** Landmark errors in millimeters between RSA and CT data and between RSA double examinations after rigid body fitting. For the RSA double examinations markers with an error of the rigid body fitting above 0.35 mm are excluded automatically by the software. This is not the case for the comparison between RSA and CT.

Volume	Mean error	Median	Standard deviation	Minimum	Maximum	95% confidence interval
*RSA-CT *						
Markers in bone	0.27	0.22	0.17	0.02	1.50	0.00–0.61
Markers in prosthesis	0.22	0.18	0.16	0.04	1.08	0.00–0.53
*RSA1-RSA2 (double examination)*						
Markers in bone	0.09	0.07	0.06	0.02	0.26	0.00–0.20
Markers in prosthesis	0.11	0.09	0.06	0.02	0.26	0.00–0.24

**(a):** 

	Rotation (degrees)			Translation (mm)			MTPM
	*X* (Transverse axis)	*Y* (Longitudinal axis)	*Z* (Sagittal axis)	*X* (Transverse axis)	*Y* (Longitudinal axis)	*Z* (Sagittal axis)	
*RSA-CT (n* = 46)							
Mean	−0.14	0.05	0.05	−0.01	−0.08	−0.04	0.30
Median	−0.02	−0.03	0.09	0.02	−0.09	−0.03	0.26
95% conf. interval (mean ± 1.96 SD)							
Lower	−1.02	−1.31	−0.56	−0.37	−0.40	−0.47	−0.02
Upper	0.75	1.21	0.65	0.34	0.24	0.39	0.61
Standard deviation (SD)	0.45	0.64	0.31	0.18	0.16	0.22	0.16
Min	−1.23	−1.58	−0.73	−0.71	−0.35	−0.47	0.10
Max	1.00	1.38	0.66	0.32	0.66	0.41	0.78

*RSA1-RSA2 (double examination)* (*n* = 46)							
Mean	0.02	−0.09	−0.04	−0.04	0.00	−0.01	0.14
Median	−0.04	−0.11	−0.02	−0.01	0.00	−0.01	0.12
95% conf. interval (mean ± 1.96 SD)							
Lower	−0.84	−0.90	−0.41	−0.29	−0.10	−0.23	−0.10
Upper	0.88	0.72	0.32	0.22	0.10	0.20	0.37
Standard deviation (SD)	0.44	0.41	0.18	0.13	0.05	0.11	0.12
Min	−0.92	−1.44	−0.80	−0.77	−0.16	−0.24	0.02
Max	2.23	0.90	0.42	0.18	0.17	0.32	0.78

**(b):** 

	Rotation (degrees)			Translation (mm)			MTPM
	*X* (Transverse axis)	*Y* (Longitudinal axis)	*Z* (Sagittal axis)	*X* (Transverse axis)	*Y* (Longitudinal axis)	*Z* (Sagittal axis)	
*RSA-CT (n* = 46)							
95% confidence limit (2.01 × SD)	0.90	1.29	0.62	0.36	0.33	0.44	0.32
Mean	−0.14	0.05	0.05	−0.01	−0.08	−0.04	0.30
Min	−1.23	−1.58	−0.73	−0.71	−0.35	−0.47	0.10
Max	1.00	1.38	0.66	0.32	0.66	0.41	0.78

*RSA1-RSA2 (double examination)* (*n* = 46)							
95% confidence limit (2.01 × SD)	0.88	0.83	0.37	0.26	0.10	0.22	0.24
Mean	0.02	−0.09	−0.04	−0.04	0.00	−0.01	0.14
Min	−0.92	−1.44	−0.80	−0.77	−0.16	−0.24	0.02
Max	2.23	0.90	0.42	0.18	0.17	0.32	0.78

**(c):** 

	Rotation (degrees)			Translation (mm)			MTPM
	*X* (Transverse axis)	*Y* (Longitudinal axis)	*Z* (Sagittal axis)	*X* (Transverse axis)	*Y* (Longitudinal axis)	*Z* (Sagittal axis)	
*RSA-CT (n* = 46)							
95% confidence limit (mean of absolute differences + 1.96 × SD)	1.23	1.78	0.86	0.48	0.46	0.61	0.61
99% confidence limit (mean of absolute differences + 2.575 × SD)	1.51	2.17	1.05	0.59	0.56	0.74	0.71

*RSA1-RSA2 (double examination)* (*n* = 46)							
95% confidence limit (mean of absolute differences + 1.96 × SD)	1.09	1.10	0.48	0.32	0.14	0.30	0.37
99% confidence limit (mean of absolute differences + 2.575 × SD)	1.36	1.36	0.60	0.40	0.17	0.37	0.45

## References

[1] Pijls B. G., Nieuwenhuijse M. J., Fiocco M. (2012). Early proximal migration of cups is associated with late revision in THA: a systematic review and meta-analysis of 26 RSA studies and 49 survival studies. *Acta Orthopaedica*.

[2] Pijls B. G., Valstar E. R., Nouta K.-A. (2012). Early migration of tibial components is associated with late revision: a systematic review and meta-analysis of 21,000 knee arthroplasties. *Acta Orthopaedica*.

[3] Selvik G. (1989). Roentgen stereophotogrammetry: a method for the study of the kinematics of the skeletal system. *Acta Orthopaedica Scandinavica. Supplementum*.

[4] Kärrholm J., Gill R. H. S., Valstar E. R. (2006). The history and future of radiostereometric analysis. *Clinical Orthopaedics and Related Research*.

[5] Kärrholm J., Herberts P., Hultmark P., Malchau H., Nivbrant B., Thanner J. (1997). Radiostereometry of hip prostheses: review of methodology and clinical results. *Clinical Orthopaedics and Related Research*.

[6] Garellick G., Rogmark C., Kärrholm J., Rolfson O. *Årsrapport 2012: För verksamhetsåret 2012*.

[7] Bottner F., Su E., Nestor B., Azzis B., Sculco T. P., Bostrom M. (2005). Radiostereometric analysis: the hip. *HSS Journal*.

[8] Derwin K. A., Milks R. A., Davidson I., Iannotti J. P., McCarron J. A., Bey M. J. (2012). Low-dose CT imaging of radio-opaque markers for assessing human rotator cuff repair: accuracy, repeatability and the effect of arm position. *Journal of Biomechanics*.

[9] Ericson A., Arndt A., Stark A. (2007). Fusion of radiostereometric analysis data into computed tomography space: application to the elbow joint. *Journal of Biomechanics*.

[10] Olivecrona H., Maguire G. Q., Noz M. E., Zeleznik M. P., Kesteris U., Weidenhielm L. (2016). A CT method for following patients with both prosthetic replacement and implanted tantalum beads: preliminary analysis with a pelvic model and in seven patients. *Journal of Orthopaedic Surgery and Research*.

[11] Sandgren B., Skorpil M., Nowik P. (2016). Assessment of wear and periacetabular osteolysis using dual energy computed tomography on a pig cadaver to identify the lowest acceptable radiation dose. *Bone and Joint Research*.

[12] Olivecrona L., Crafoord J., Olivecrona H. (2002). Acetabular component migration in total hip arthroplasty using CT and a semiautomated program for volume merging. *Acta Radiologica*.

[13] Olivecrona H., Olivecrona L., Weidenhielm L. (2003). Stability of acetabular axis after total hip arthroplasty, repeatability using CT and a semiautomated program for volume fusion. *Acta Radiologica*.

[14] Olivecrona L., Olivecrona H., Weidenhielm L., Noz M. E., Maguire G. Q., Zeleznik M. P. (2003). Model studies on acetabular component migration in total hip arthroplasty using CT and a semiautomated program for volume merging. *Acta Radiologica*.

[15] Olivecrona H., Weidenhielm L., Olivecrona L. (2004). A new CT method for measuring cup orientation after total hip arthroplasty: a study of 10 patients. *Acta Orthopaedica Scandinavica*.

[16] Seehaus F., Olender G. D., Kaptein B. L., Ostermeier S., Hurschler C. (2012). Markerless Roentgen Stereophotogrammetric Analysis for in vivo implant migration measurement using three dimensional surface models to represent bone. *Journal of Biomechanics*.

[17] Bonel H. M., Jäger L., Frei K. A. (2005). Optimization of MDCT of the wrist to achieve diagnostic image quality with minimum radiation exposure. *American Journal of Roentgenology*.

[18] Mäkinen T. J., Koort J. K., Mattila K. T., Aro H. T. (2004). Precision measurements of the RSA method using a phantom model of hip prosthesis. *Journal of Biomechanics*.

[19] Sint Jan S. V., Sobzack S., Dugailly P.-M. (2006). Low-dose computed tomography: a solution for in vivo medical imaging and accurate patient-specific 3D bone modeling?. *Clinical Biomechanics*.

[20] Oka K., Murase T., Moritomo H., Goto A., Sugamoto K., Yoshikawa H. (2009). Accuracy analysis of three-dimensional bone surface models of the forearm constructed from multidetector computed tomography data. *The International Journal of Medical Robotics and Computer Assisted Surgery*.

[21] Otten V. T. C., Crnalic S., Röhrl S. M., Nivbrant B., Nilsson K. G. (2016). Stability of uncemented cups—long-term effect of screws, pegs and HA coating: a 14-year RSA follow-up of total hip arthroplasty. *Journal of Arthroplasty*.

[22] Charnley J. (1972). The long-term results of low-friction arthroplasty of the hip performed as a primary intervention. *Journal of Bone and Joint Surgery B*.

[23] Röhrl S. M., Nivbrant B., Ström H., Nilsson K. G. (2004). Effect of augmented cup fixation on stability, wear, and osteolysis: a 5-year follow-up of total hip arthroplasty with RSA. *The Journal of Arthroplasty*.

[24] Gallo J., Havranek V., Zapletalova J. (2010). Risk factors for accelerated polyethylene wear and osteolysis in ABG I total hip arthroplasty. *International Orthopaedics*.

[25] Bongartz G., Golding S., Jurik A. (2000). European guidelines on quality criteria for computed tomography. *EUR*.

[26] Servomaa A., Tapiovaara M. (1998). Organ dose calculation in medical X ray examinations by the program PCXMC. *Radiation Protection Dosimetry*.

[27] Valstar E. R., Gill R., Ryd L., Flivik G., Börlin N., Kärrholm J. (2005). Guidelines for standardization of radiostereometry (RSA) of implants. *Acta Orthopaedica*.

[28] Noz M. E., Maguire G. Q., Zeleznik M. P., Kramer E. L., Mahmoud F., Crafoord J. (2001). A versatile functional-anatomic image fusion method for volume data sets. *Journal of Medical Systems*.

[29] Scheerlinck T., Polfliet M., Deklerck R., Van Gompel G., Buls N., Vandemeulebroucke J. (2016). Development and validation of an automated and marker-free CT-based spatial analysis method (CTSA) for assessment of femoral hip implant migration In vitro accuracy and precision comparable to that of radiostereometric analysis (RSA). *Acta Orthopaedica*.

[30] Svedmark P., Lundh F., Németh G. (2011). Motion analysis of total cervical disc replacements using computed tomography: preliminary experience with nine patients and a model. *Acta Radiologica*.

[31] Söderkvist I., Wedin P.-Å. (1993). Determining the movements of the skeleton using well-configured markers. *Journal of Biomechanics*.

[32] Bland J. M., Altman D. G. (1986). Statistical methods for assessing agreement between two methods of clinical measurement. *The Lancet*.

[33] R Core Team *R: A Language and Environment for Statistical Computing*.

[34] Ranstam J., Ryd L., Onsten I. (2000). Accurate accuracy assessment: review of basic principles. *Acta Orthopaedica Scandinavica*.

[35] Sköldenberg O., Eisler T., Stark A., Muren O., Martinez-Carranza N., Ryd L. (2014). Measurement of the migration of a focal knee resurfacing implant with radiostereometry. *Acta Orthopaedica*.

[36] Malchau H., Kärrholm J., Wang Y. X., Herberts P. (1995). Accuracy of migration analysis in hip arthroplasty. Digitized and conventional radiography, compared to radiostereometry in 51 patients. *Acta Orthopaedica Scandinavica*.

[37] Kärrholm J. (1989). Roentgen stereophotogrammetry: review of orthopedic applications. *Acta Orthopaedica*.

[38] Ryd L., Yuan X., Löfgren H. (2000). Methods for determining the accuracy of radiostereometric analysis (RSA). *Acta Orthopaedica Scandinavica*.

[39] Gurung J., Khan M. F., Maataoui A. (2005). Multislice CT of the pelvis: dose reduction with regard to image quality using 16-row CT. *European Radiology*.

[40] Ericson A., Olivecrona H., Stark A. (2010). Computed tomography analysis of radiostereometric data to determine flexion axes after total joint replacement: application to the elbow joint. *Journal of Biomechanics*.

